# Supplementary material for: RyR2/IRBIT regulates insulin gene transcript, insulin content, and secretion in the insulinoma cell line INS-1
Source: Sci Rep. 2022 May 11;12:7713. doi: 10.1038/s41598-022-11276-8 (PMC9095623; doi:10.1038/s41598-022-11276-8)
Supplement: Supplementary file 1 — Supplementary Information. [file 41598_2022_11276_MOESM1_ESM.docx]

**Supplementary Materials**


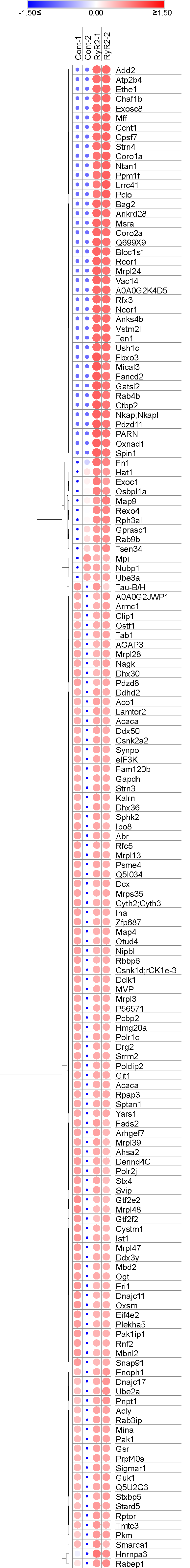


**Figure S1. Proteins with increased abundance in RyR2^KO^ cells compared to control INS-1 cells as determined by LC-MS/MS**


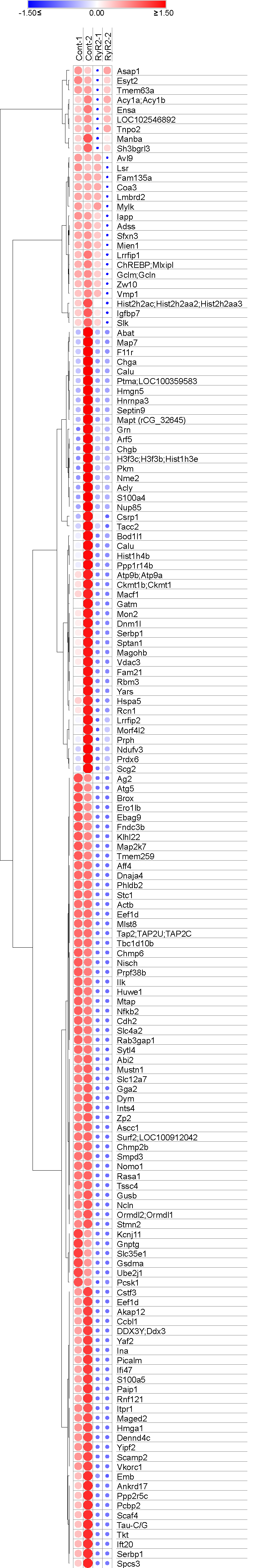


**Figure S2. Proteins with decreased abundance in RyR2^KO^ cells compared to control INS-1 cells as determined by LC-MS/MS**


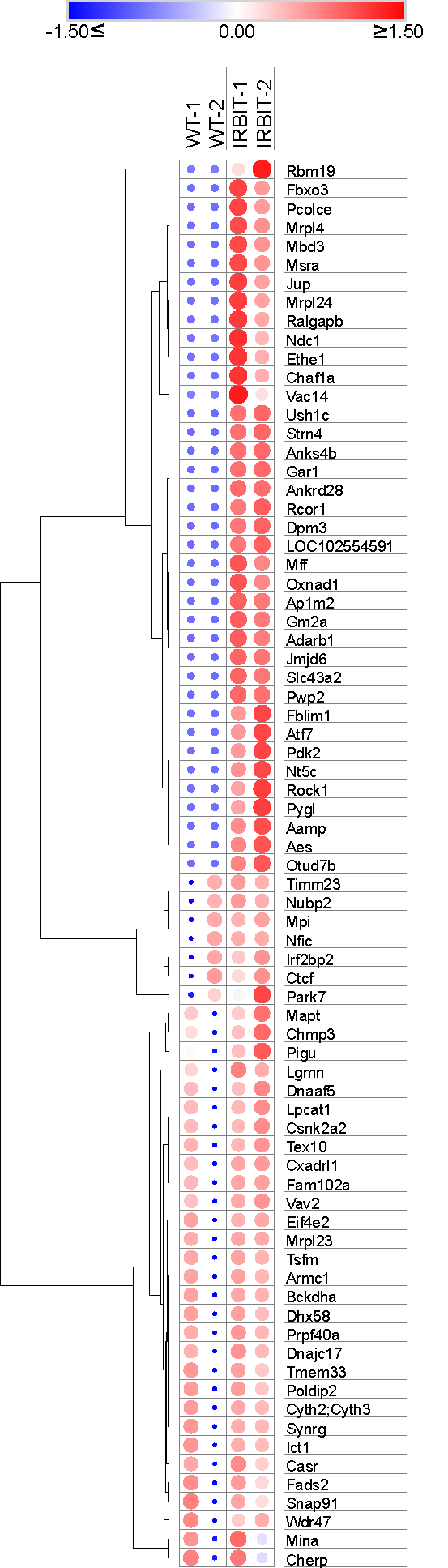


**Figure S3. Proteins with increased abundance in IRBIT^KO^ cells compared to control INS-1 cells as determined by LC-MS/MS.**


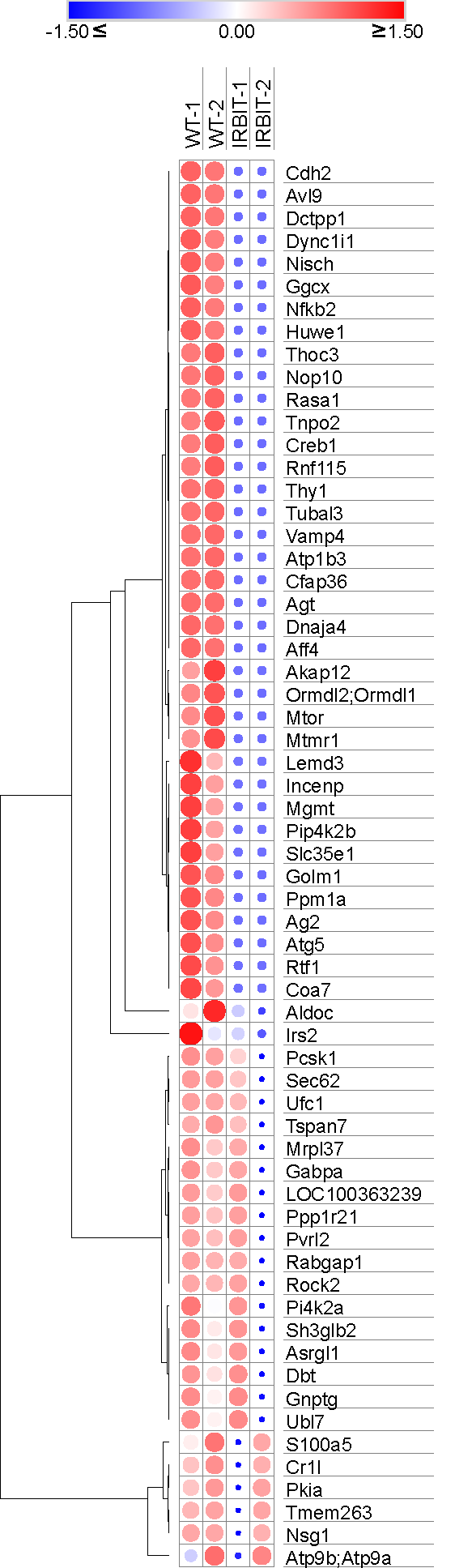


**Figure S4. Proteins with reduced abundance in IRBIT^KO^ cells compared to control INS-1 cells as determined by LC-MS/MS.**


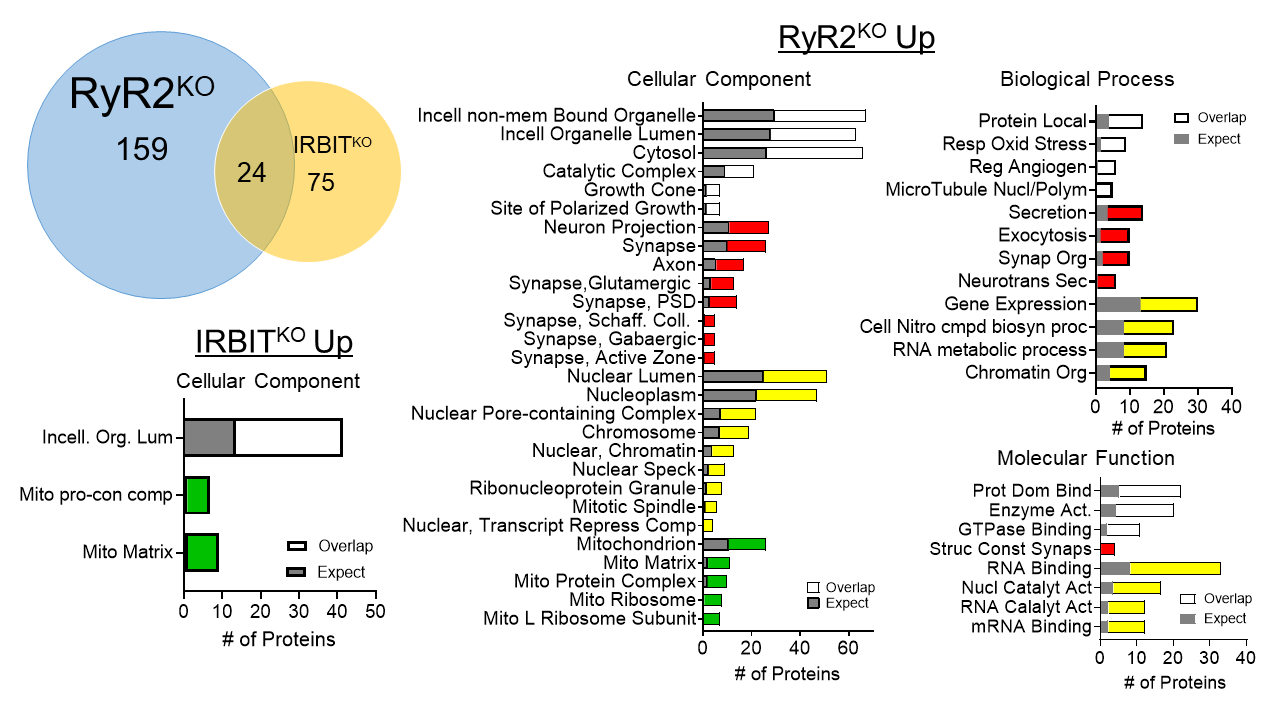


**Figure S5. GO analysis of up-regulated proteins in RyR2^KO^ and IRBIT^KO^ cells-** The number of proteins with increased abundance in RyR2^KO^ or IRBIT^KO^ cells that belong to each of the indicated GO categories (overlap- shown as white, red, yellow, or green bars) is indicated, along with the expected number of proteins in each category (gray bars). Red bars indicate neuronal protein categories, yellow bars indicate nuclear protein categories, and green bars indicate mitochondrial protein categories. FDR < 0.05; overrepresentation > 2-fold.


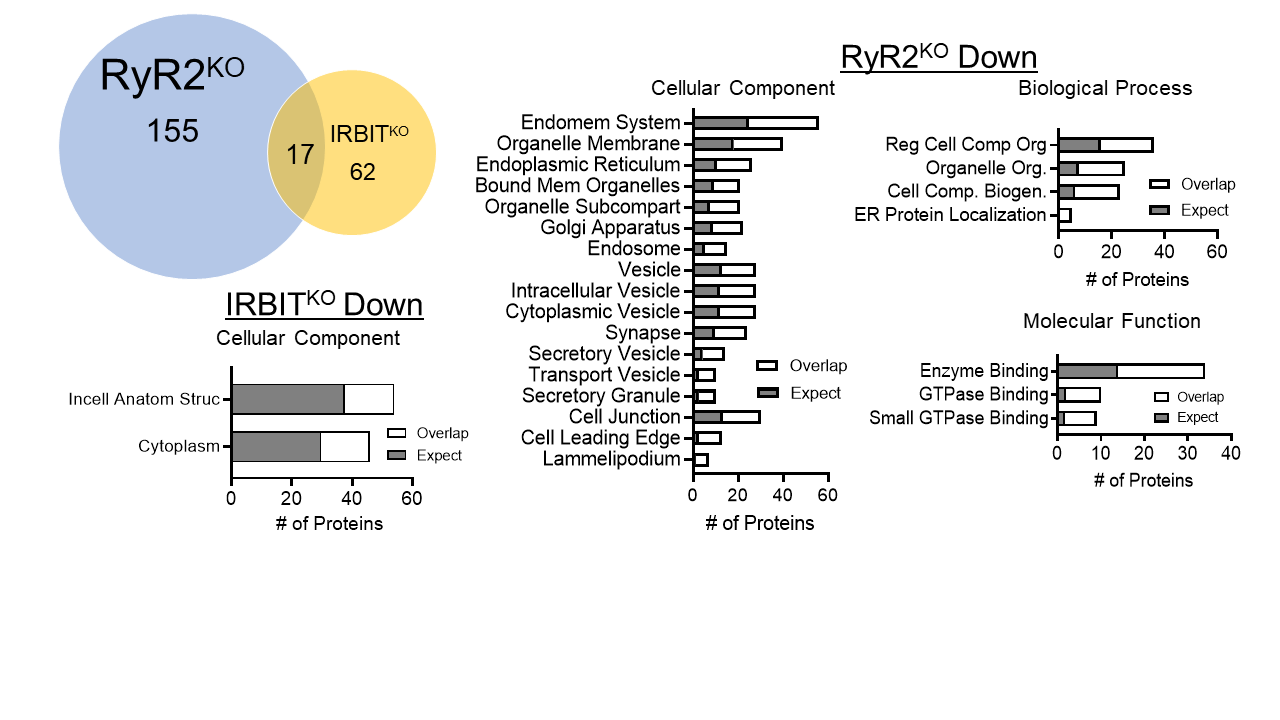


**Figure S6. GO analysis of down-regulated proteins in RyR2^KO^ and IRBIT^KO^ cells-** The number of proteins with decreased abundance in RyR2^KO^ cells that belong to each of the indicated GO categories (overlap- shown as white) is indicated, along with the expected number of proteins in each category (gray bars). FDR < 0.05.


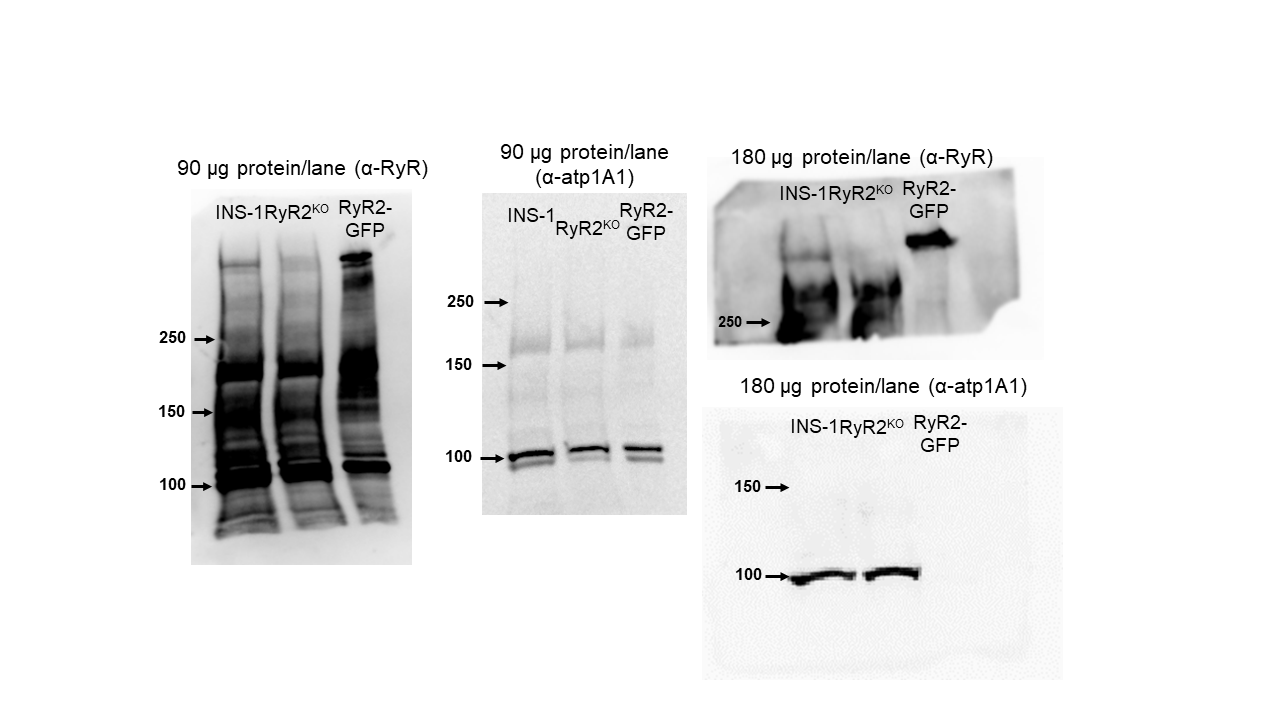


**Figure S7. Full-length immunoblots from Figure 1e-** Arrows indicate the position of a molecular weight marker, given in kDaltons.


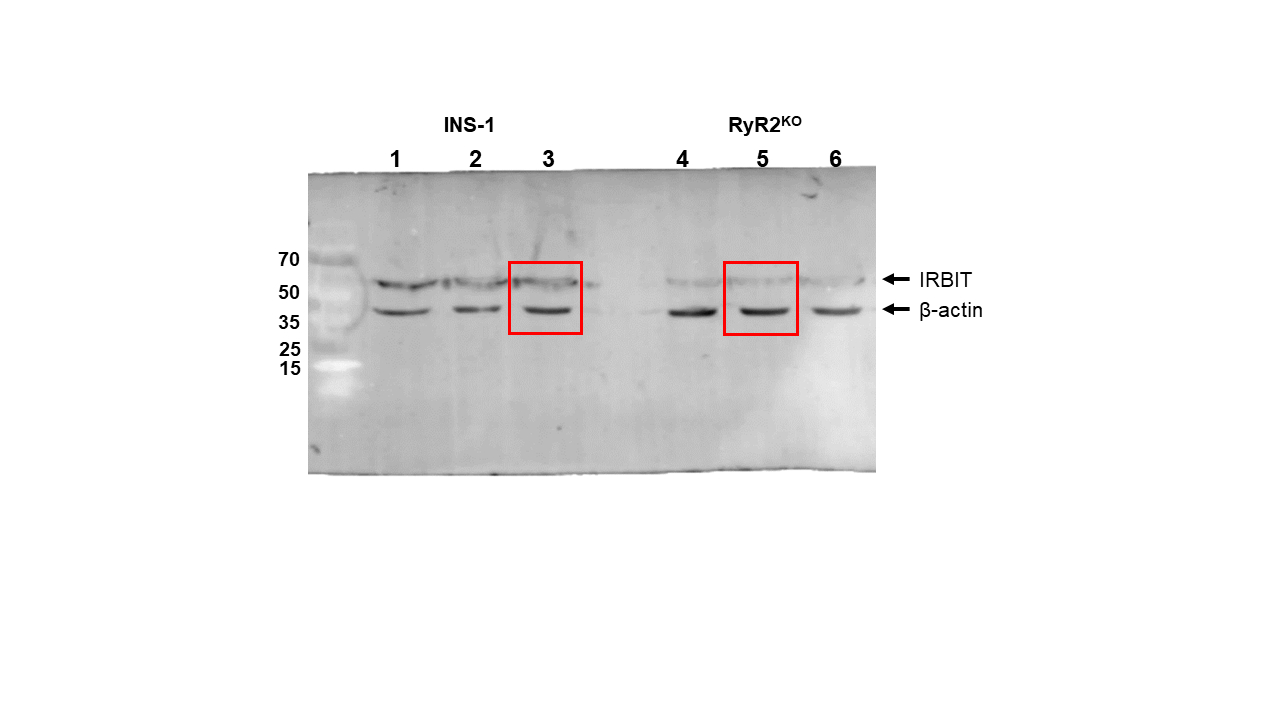


**Figure S8. Full-length immunoblots from Figure 3e-** Numbers indicate molecular weight (in kDa) of markers visible on the left edge of the membrane. Lanes 3 and 5 (red boxes) were juxtaposed to create Figure 3e.

**Figure S9. Full-length immunoblots from Figure 4b-** Arrows indicate the position of molecular weight markers, given in kDa. Red boxes indicate areas that were cropped and included in Figure 4b.


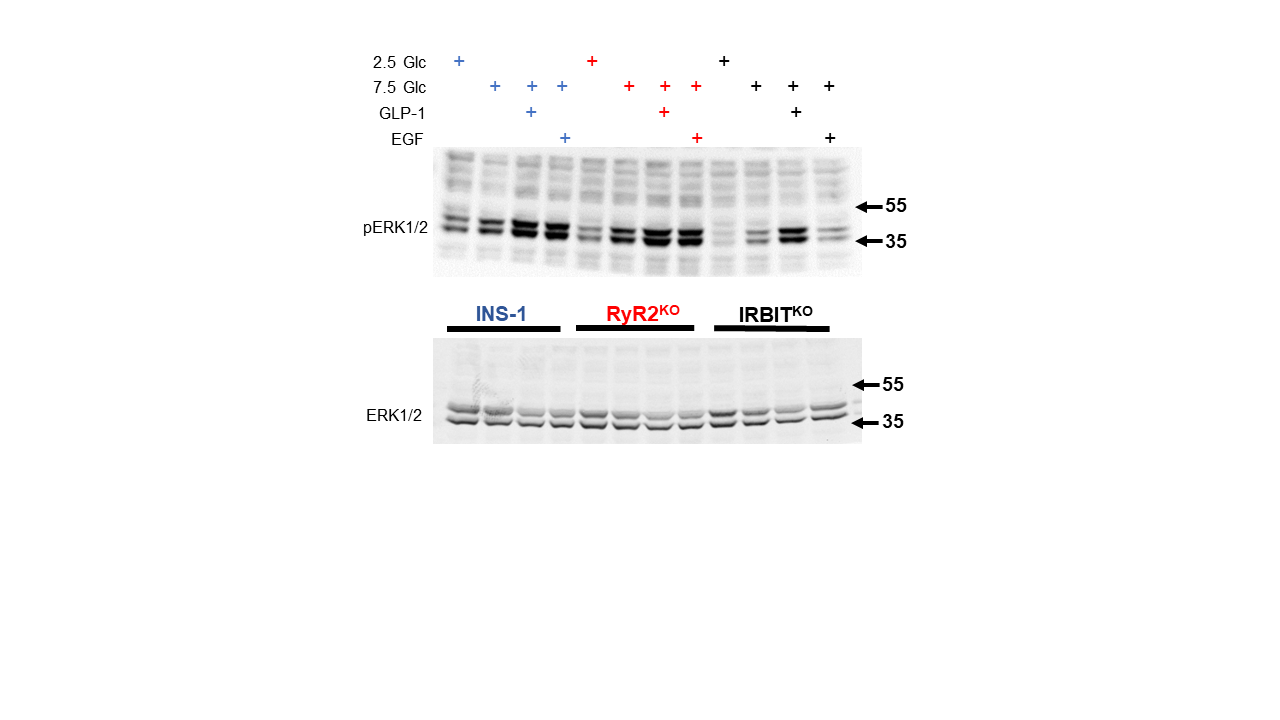


**Figure S10. Full-length immunoblots from Figure 5d-** Arrows indicate the position of molecular weight markers, given in kDa. Top panel was blotted with a phospho-specific ERK1/2 antibody. Bottom panel was blotted with a non-phospho-specific ERK1/2 antibody.
